# The Unit and Scale-Up Cost of Postabortion Care in Tanzania

**DOI:** 10.9745/GHSP-D-19-00035

**Published:** 2019-08-22

**Authors:** Colin Baynes, Erick Yegon, Godfather Kimaro, Grace Lusiola, Justin Kahwa

**Affiliations:** a EngenderHealth, New York, NY, USA.; b EngenderHealth, Nairobi, Kenya.; c The National Institutes of Medical Research Tanzania, Dar es Salaam, Tanzania.; d EngenderHealth, Dar es Salaam, Tanzania.

## Abstract

**Introduction:**

Based on research conducted in 2017, we estimated the cost to the Tanzanian health care system of providing postabortion care (PAC). PAC is an integrated service package that addresses the curative and preventive needs of women experiencing complications from abortion. PAC services include treating complications of miscarriage and incomplete abortion, providing voluntary family planning counseling and services, and engaging the community to reduce future unintended pregnancies and repeat abortions.

**Methods:**

Thirty-one public and private health facilities, representing 3 levels of health care, were selected for data collection from key care providers and administrators in 3 regions. We gathered data on the direct costs of PAC startup (i.e., training and capital costs), as well as the recurrent costs of medicines, supplies, hospitalization, and personnel, and the indirect costs of PAC provision. We also gathered data to estimate PAC clients’ out-of-pocket expenses. Estimates of the average cost per client (i.e., unit cost) were calculated for treatment of routine and severe abortion complications, treatment at different levels of health care, postabortion contraception, and various available treatment methods.

**Results:**

We found that the unit cost of PAC training per provider was US$163.43. The total unit cost was $72.91. The unit recurrent cost of treating routine complications, which included 81% of the cases in our sample, was $36.23. The cost of treating incomplete abortion through manual vacuum aspiration was $22.63, while the cost of treatment with misoprostol was $18.74. The average cost of providing voluntary postabortion family planning was $11.56. We estimated an average client out-of-pocket expenditure on PAC of $22.96.

**Conclusion:**

We applied our unit cost estimates to those on PAC utilization and provision and unmet need for PAC that were derived from research conducted in Tanzania in 2013–2016, and we estimated an annual national cost of PAC of $4,170,476. We estimated the cost of providing PAC for all women who have abortion complications, including those who do not access PAC, at $10,426,299. Investing more resources in voluntary family planning and PAC treatment of routine complications at the primary level would likely reduce health system costs.

## Abstract (precis)

Given the high burden and cost of postabortion care (PAC) in Tanzania, health policy should strengthen voluntary family planning programs and the availability of a variety of contraceptive methods to PAC clients. A particular focus should be placed on decentralizing PAC to lower-level facilities, including health centers and dispensaries, which can provide safe, accessible, and appropriate PAC at the lowest cost including surgical or medical options.

## INTRODUCTION

Deaths of women from complications of pregnancy or childbirth are unacceptably high in Tanzania. By 2016, the maternal mortality ratio (556 deaths per 100,000 live births) had changed little from the preceding decade.^1^ Complications arising from abortion—regardless of whether it is spontaneous or induced—substantially contribute to this burden of preventable suffering in Tanzania. The little evidence available for Tanzania, which is mainly from small-scale hospital-based studies, suggests that abortions are widespread, largely unsafe, and associated with high morbidity and mortality. According to these studies, slightly over 60% of women admitted to a hospital for a miscarriage had had an induced abortion.^2^^–^^4^ One study indicated that complications from abortion account for 38% of hospitalizations for obstetric complications.^5^ Two other hospital studies reported that complications from abortion account for approximately one-quarter of maternal deaths.^6^^,^^7^

Caring for women that require treatment for abortion complications is often expensive in terms of skilled personnel, surgical procedures, drugs and supplies, and hospitalization.^8^^–^^10^ Across countries, the financial burden of postabortion care (PAC) is considerable. Studies in Ethiopia, Rwanda, and Uganda suggested that treating abortion complications consumes between 4% and 6% of total expenditures on reproductive health in these countries.^11^^–^^13^ In Uganda and Rwanda, such treatment accounts for 29% and 11%, respectively, of annual per capita income.^14^ Prior research from this region has identified strategies that might reduce health care costs with regard to PAC. Studies in Uganda and Malawi found that the mean health facility cost per case of PAC could be reduced by 43% by shifting from the use of dilation and curettage for incomplete abortion to manual vacuum aspiration (MVA), which the World Health Organization (WHO) recommends, and by increasing the proportion of PAC cases handled by midlevel providers and low-level health centers.^15^^,^^16^

Across countries, the financial burden of PAC is considerable.

Although no formal studies have estimated the cost of PAC in Tanzania, Keogh and colleagues^17^ reported valuable information on the incidence of abortion and utilization of PAC in the country. Specifically, they estimated the annual number of abortions to be 405,000 (36 per 1,000 women of reproductive age) and a ratio of 1:6 with regard to the number of women that access PAC versus the number who had abortions and may have required the service. In mainland Tanzania, the Ministry of Health, Community Development, Gender, Elderly, and Children has authorized the provision of surgical PAC services using sharp curettage and MVA, but not medical treatment of incomplete abortion using misoprostol, a uterotonic drug that women can take orally or sublingually. Zanzibar, which has its own Ministry of Health, permits MVA, sharp curettage, and misoprostol for PAC. According to global recommendations, for PAC treatment of incomplete abortion, sharp curettage should be phased out and replaced with vacuum aspiration and medical methodologies.^18^ All women who receive PAC are eligible to choose from a wide range of contraceptive methods immediately after their treatment procedure, regardless of its type.^19^

We conducted a study in 2017 in Arusha and Mwanza regions in mainland Tanzania and in Zanzibar, a semiautonomous island region of the country, to obtain detailed cost estimates of the unit and health system costs of PAC provision in these settings. By applying unit cost calculations to earlier estimations of PAC utilization and unmet need for treatment for incomplete abortion, we estimated the current health system cost of PAC and the cost of ensuring access for all women needing treatment for incomplete abortion, including those who do not access it.

We estimated the current health system cost of PAC in Tanzania and the cost of ensuring access for all women who need it.

## METHODS

The data used for this study were collected at 31 health facilities between June and September 2017. These facilities were selected from a sample frame of 176 facilities that were providing PAC and had enrolled in projects led by EngenderHealth, an international women’s health NGO working to strengthen the integration of emergency treatment for postabortion complications and the provision of a wide range of voluntary contraceptive methods. Our sample frame included facilities representing different levels of care of Tanzania’s health care system (regional and district hospitals, intermediate health centers, and primary-level dispensaries) and ownership structures (public and private) in 3 regions of the country—Arusha, Mwanza, and Zanzibar. At the tertiary level, we selected all referral hospitals (n=4) and randomly selected 9 district hospitals, at least 1 in each region. Stratifying by regions, we randomly chose 13 health centers and 5 dispensaries, ensuring that we had an adequate balance between rural and urban facilities. Because of cost and time considerations, collecting data from more facilities or a greater number of regions was not possible. Sensitivity analyses were used to test for variability in our estimates of direct medical costs, capital and indirect costs, percent distribution of PAC cases by facility type, and percent distribution of cases by complication type. All estimates reported in this analysis pertain to economic costs, rather than financial costs.

Prior to commencing the research, we obtained clearance letters from the National Ethics Committees in both the mainland and in Zanzibar. All potential participants were read an informed consent form, and all participants signed the document before data collection began. Information compiled from participants emphasized startup costs for establishing PAC services in terms of training and infrastructure (i.e., capital costs); recurrent costs, including those associated with medicines, supplies, commodities, personnel, and hospitalization (i.e., direct costs); and indirect costs. These inputs collectively compose the health system cost of PAC (i.e., treatment of complications and voluntary family planning services). In addition, we randomly sampled PAC clients immediately after they were discharged from care to obtain information on out-of-pocket (OOP) expenditures. Costs were further broken down according to the 5 major abortion complications included in the WHO study on costing in its “Mother Baby Package”: incomplete abortion, shock, sepsis, vaginal or cervical laceration, and perforation of the uterus or lower abdomen.^20^

A primary feature of the methodology used for obtaining data on recurrent and capital costs is its reliance on a key informant approach to estimation. The respondents were experts knowledgeable about PAC through years of experience at the facilities where our study took place. They included medical officers in charge of health facilities, hospital health secretaries, PAC and family planning providers (namely, midwives, nurses, and clinical officers), matrons (i.e., nurses in-charge), anesthetists, operating theater nurses, laboratory providers and managers, and health management information personnel. For costs related to medicines and supplies and hospitalization, key informants estimated the total number of PAC clients admitted at their respective facilities by complication type during 2016 and whether they were hospitalized or seen as outpatients. For administrative questions, such as salaries and overhead expenditures, we interviewed health administrators and district and hospital levels. Area managers and engineers were also interviewed in order to gather information on capital costs. Respondents were asked to provide estimates of the many detailed inputs that, in total, constitute a complete intervention for treating the respective abortion complications. We assumed that the respondents’ estimates would on average yield a good approximation of the true values of the various rates and amounts of specific inputs. Overall, 124 interviews were conducted with key informants to obtain data on startup and recurrent costs.

Information on 2016 PAC training expenditures—items, their quantities, and amounts spent—at the 176 sites that composed the sampling frame were obtained from 3 sources: facilities-in-charges, who reported on the numbers of staff trained in PAC provision; project activity expenditure records of the EngenderHealth program that facilitated these trainings; and interviews with EngenderHealth staff who provided necessary clarifications. To estimate the cost of carrying out these same activities through the PAC training operations of the Ministry of Health, Community Development, Gender, Elderly and Children, we carried out in-depth interviews with management staff and financial officers, reviewed available expenditure records for the same items and quantities identified for the EngenderHealth trainings at government training centers in the respective regions. With this information, we determined the costs of the training health care workers in PAC at public-sector training institutions. Information on capital (infrastructure) and recurrent costs used for PAC was collected from a questionnaire that was divided into 5 sections, one for each abortion complication for which PAC clients were treated. The questionnaire gathered data on inputs needed for estimating the cost of treating each complication, respectively, including information on personnel inputs of time, personnel wages, hospitalization, and capital costs. Detailed data on the quantities of all medicines, commodities, and supplies used in specific postabortion treatments were also collected. The questionnaire collected information separately for treating inpatient and outpatient cases. For each complication, several detailed probes solicited information on (1) the percentage of patients with a particular complication who needed the specific input, and (2) the input that an average patient was given during the full course of treatment. The reference year for this study’s estimates was 2016, and all costs are given in US dollars from that year. These costs were converted from Tanzanian shillings (TSH) based on the average exchange rate for 2016 (US$1=2,186 TSH).

Lastly, the study gathered information from PAC clients on the OOP expenses they incurred to access PAC. These clients were consecutively sampled at certain facilities chosen from among the 31 sites where the other data were collected. PAC clients reported OOP costs on travel, hospitalization, food, medicines and supplies, laboratory tests, and any other expenditures they made to access PAC. This information was obtained from 25 PAC clients at 16 facilities in Arusha, Mwanza, and Zanzibar regions.

This study also drew upon research carried out by the Guttmacher Institute in Tanzania between 2013 and 2016, which estimated that 405,000 women in Tanzania have abortions annually and that for each woman treated in a facility for abortion complications, 6.08 women had an abortion but did not receive PAC.^17^ This lack of PAC might be because they did not have complications or because their complications were untreated. The Guttmacher Institute also reported that only 40% of women who have abortion complications receive PAC.^21^ Based on this information, we estimated out of all women who have abortions, 143,009 women experience abortion complications and require medical care, of whom 57,203 receive PAC.

## RESULTS

We first present the details of our sample of sites and cases, based on data collected in 2017, which reflect cases and inputs estimated for 2016. We then present estimates of the startup costs on training, per health worker trained, and capital expenditures, overall and per client in 2016 (N=6,336). Unit costs (average cost per client) per category of recurrent expenditure and overall are then presented and explained. We then apply our estimate of the unit cost to the estimated 57,203 women who are able to receive PAC each year. We also estimate the cost of treating all women expected to experience abortion complications each year, including those who do not access PAC.

### Characteristics of PAC Cases Estimated

Table 1 presents the total number of PAC cases estimated by key informants. Across all facilities and complications, the informants conjectured that 6,336 women received PAC in 2016, of whom nearly two-thirds (65%; n=4,092) were hospitalized. Among the women receiving PAC, incomplete abortions accounted for 81% of all treatments for abortion complications (n=5,163); 7% each for sepsis (n=475) and shock (n=453); 3% for lacerations (n=188); and 1% for perforations (n=57). Overall, 42% of cases were managed at regional hospitals (n=2,678) and 34% at district hospitals (n=2,178). Less than a quarter of cases were managed at the primary level, with 20% at health centers (n=1,253) and 4% at dispensaries (n=227).

**TABLE 1.** Number of Postabortion Cases Estimated by Facility Type by Complication and Hospitalization Status During Data Collection, June–September 2017

	Type of Complication and Hospitalization Status													
	Incomplete Abortion		Shock		Sepsis		Laceration		Uterine Perforation		All Complications		Total	
Facility Type	H	OP	H	OP	H	OP	H	OP	H	OP	H	OP	H	OP
Regional hospital	1,881	286	228	20	216	7	11	7	18	4	2,354	324	88%	12%
District hospital	940	859	114	1	129	0	21	95	16	3	1,220	958	56%	44%
Health center	296	695	89	2	82	20	41	12	10	6	518	735	41%	59%
Dispensary	NA	206	NA	0	NA	21	NA	0	NA	0	NA	227	0%	100%
Total	3,117	2,046	431	23	427	48	73	114	44	13	4,092	2,244	65%	35%
%	60%	40%	95%	5%	90%	10%	39%	61%	77%	23%	65%	35%		

Abbreviations: H, hospitalized; NA, not applicable; OP, outpatient.

Key informants estimated that more than 6,000 women received postabortion care in 2016.

Regarding cases of incomplete abortion, informants estimated that 84% (n=4,340) of PAC clients were treated with 1 of 3 treatment methods (MVA, misoprostol, or sharp curettage) and 16% (n=823) treated by way of expectant management. Of those who received treatment for incomplete abortion, 64% (n=2,759) were treated with MVA, 17% (n=737) with misoprostol, and 19% (n=844) with sharp curettage. Of all PAC clients, no matter the complication, informants estimated that 73% (n=4,643) were discharged from PAC with a voluntary modern contraceptive method of whom 52% (n=2,414) received a short-acting method and 48% (n=2,229) received a long-acting reversible method (LARC) or permanent method (Box 1).

BOX 1. **Key Informants’ Estimates of Key Postabortion Care Indicators**

- Almost two-thirds of postabortion care clients are hospitalized for more than 24 hours.
- Almost one-fifth are admitted for a severe complication (sepsis, shock, cervical/vaginal laceration, uterine perforation).
- Roughly three-quarters of postabortion care clients are seen at tertiary facilities (regional and district hospitals).
- Of clients admitted for routine complications from incomplete abortion, almost one-fifth receive sharp curettage.
- Of clients admitted for routine complications from incomplete abortion, nearly three-quarters receive a voluntary contraceptive method.

### Startup Costs for PAC Provision

#### Training

We estimated the cost of training 352 providers from the 176 sites in Arusha, Mwanza, and Zanzibar in 2016. These sites constituted the frame we used for sampling the 31 sites used for the other components of our study. Our estimates of the training cost per provider vary significantly between those calculated for the mainland sites, where we estimated an average cost of training per provider of $125.30, and sites in Zanzibar, where our estimate was $201.56. Reasons for this discrepancy relate to differences in training capacity. Unlike Arusha and Mwanza, which have long-standing PAC programs that include established zonal training centers, for trainings in Zanzibar, national trainers from the mainland are needed to facilitate training activities. Of note, we also analyzed the cost of supplementing essential training in PAC (which includes postabortion treatment, counseling on complications management, and provision of voluntary family planning) with technical training on the insertion and removal of LARC methods. This analysis was done in accordance with the government of Tanzania’s recent decisions to integrate training in these methods in the curricula on the essential elements of PAC.^22^ We found that integrating LARCs into essential PAC training substantially increased the average training cost, by $517.24 per provider in mainland and $856.64 per provider in Zanzibar.

Our estimates of the training cost per provider vary significantly between mainland sites and sites in Zanzibar.

#### Capital Costs

Capital costs include expenditure on construction and health facility equipment, including operating theater costs, required for PAC provision. Since these costs are essential for initiating services but are amortized gradually over time, we considered capital costs as startup investments. We also considered them to be a key expenditure to include in the unit health system cost estimations of PAC, mindful that PAC is typically provided as an outpatient service, not in operating theaters. In our analysis, we estimated capital costs from inputs having more than 1 year of useful life, and we annuitized these inputs over that period. The mean number of years of useful life for inputs included in the estimation of capital costs was 28 years. Based on a literature review, we applied a discount rate of 3% per year.^23^ We found that the total cost on amortized annual constructions and equipment was $85,209.47. However, this figure was highly skewed and was significantly larger with the level of facility, ranging from $84,304 per annum at regional hospitals (n=3) to $604.18 at district hospitals (n=5), $264.76 at health centers (n=14), and $36.45 at primary-level dispensaries (n=9). The unit capital cost per PAC client was $13.46 (N=6,336).

Estimated capital costs for PAC varied widely based on level of facility, from about $84,000/year at regional hospitals to $36 at primary-level dispensaries.

The large differences between estimated capital costs at regional hospitals and lower-level facilities, respectively, relate to several factors. First, regional hospitals are referral facilities for all facilities in their regions, including district hospitals. Their mandate to provide advanced health care services, primarily by medical specialists, entails the need for expenditure on maintaining and equipping treatment settings, such as operating theaters, with expensive equipment, including ultrasound, X-ray machines, and laboratory equipment, which lower-level facilities in Tanzania typically do not have. The size of regional hospitals and the need to equip them for more frequent and longer hospitalizations relative to lower-level facilities may drive up capital costs on equipment. Regional hospitals also maintain larger ambulance fleets for emergency transport compared to lower-level facilities, and thus they incur higher capital expenditures for the purchase, maintenance, and refueling of these vehicles.

### Recurrent Costs for PAC Provision

We estimated the recurrent cost of PAC provision by complication for the following categories: personnel, medicines and supplies, and hospitalization.

#### Personnel

Several factors were used in calculating personnel costs: the percentage of cases that needed the attention of each category of health worker; the number of minutes health personnel spent attending to patients, including an upward adjustment for time related to direct service provision; and salaries, allowances, and benefits. The average number of minutes that separate cadres of health workers spent attending the different abortion complications is presented in Table 2. Treating incomplete abortion (average of 22.5 minutes per cadre) required the least input of labor, whereas treatment of uterine perforation needed the most (average of 58.3 minutes per cadre). For all complications, enrolled nurses spent the most time on managing PAC clients (51.6 minutes), followed by anesthetists and medical officers (42.7 and 42.6 minutes, respectively).

**TABLE 2.** Average Time Spent on Each Type of Postabortion Complication by Health Worker Category

	Time Spent (minutes) per Complication					
Health Worker	Incomplete Abortion	Shock	Sepsis	Laceration	Uterine Perforation	All Complications
Obstetrician/gynecologist	11.3	190.0	47.5	41.3	130.0	26.1
Anesthesiologist	0.0	0.0	3.3	7.5	20.0	9.7
Anesthetist	11.3	16.7	13.3	35.0	102.5	42.7
Anesthetist assistant	10.0	15.0	10.0	30.0	100.0	38.0
Doctor/medical officer	30.0	133.8	96.3	53.8	120.0	42.6
Assistant medical officer	26.3	10.0	3.3	3.8	5.0	23.2
Clinical officer	30.6	2.5	24.0	3.8	5.0	28.5
Registered nurse/principal enrolled nurse	23.6	94.0	123.5	66.3	25.8	39.3
Enrolled nurse (nurse midwife)	101.5	41.3	39.2	66.3	31.3	51.6
Medical attendant	25.3	20.0	55.3	20.0	86.7	24.1
Laboratory technician	22.5	12.6	16.4	13.8	35.0	19.0
Laboratory assistant	10.0	38.8	26.7	10.0	10.0	12.4
Sonographer	14.5	31.3	10.8	5.0	102.0	12.4
Pharmacist	11.3	13.3	22.5	18.8	75.0	7.7
Drug dispenser	8.8	23.3	6.3	13.8	26.3	8.4

In Table 3, we present the personnel unit costs by type of complications and category of health care worker. As with type of complication, we found that the personnel costs for nurse-midwives accounted for a significantly larger proportion of total costs on personnel than personnel costs of other workers for all complication types.

**TABLE 3.** Distribution of Personnel Costs of Postabortion Care per Health Worker and Complication Type

	% of Total Personnel Costs per Complication Type					
Health Worker	Incomplete Abortion	Shock	Sepsis	Laceration	Uterine Perforation	All Complications
Obstetrician/gynecologist	3.6	58.4	14.8	16.7	13.4	9.4
Anesthesiologist	0.0	0.0	0.0	0.0	0.0	0.0
Anesthetist	2.5	0.6	3.0	4.6	5.7	2.1
Anesthetist assistant	2.5	1.0	2.3	5.2	3.8	1.7
Doctor/medical officer	15.1	6.5	17.4	14.7	7.2	14.8
Assistant medical officer	4.7	2.2	2.8	3.1	4.3	4.7
Clinical officer	0.8	0.6	1.8	2.0	2.1	0.8
Registered nurse/principal enrolled nurse	9.7	3.8	12.4	8.3	5.7	9.2
Enrolled nurse (nurse midwife)	42.8	21.9	29.7	39.0	33.7	41.0
Medical attendant	7.4	1.6	4.1	3.6	3.2	5.7
Laboratory technician	3.1	0.6	4.1	0.6	3.0	3.0
Laboratory assistant	1.8	0.3	0.8	0.6	2.6	1.6
Sonographer	2.6	2.1	1.9	0.0	9.5	2.7
Pharmacist	2.3	0.2	4.5	1.1	5.3	2.4
Drug dispenser	1.2	0.1	0.4	0.4	0.4	1.0
Total for all workers	100.0	100.0	100.0	100.0	100.0	100.0

We estimated that the overall average cost on personnel was $18.01. However, the cost varied considerably by complication type, from $15.06 and $15.85 per case of laceration and incomplete abortion, respectively, to $53.13 per case of uterine perforation. The first panel of Table 4 presents this information by type of complication and level of health care. The range of average personnel costs was significantly skewed upward; at the regional hospitals, which admitted 2,678 PAC clients, the average cost on labor was $28.33, over 2 times as much as such costs at district hospitals and lower-level facilities (health centers and dispensaries combined).

**TABLE 4.** Average Costs of Direct Inputs for Personnel, Medicines and Supplies, and Hospitalization for PAC Provision by Type of Complication (US$ in 2016)

	Type of Complication					
	Incomplete Abortion	Shock	Sepsis	Laceration	Uterine Perforation	All Complications
**Average cost of personnel used in PAC treatment**						
Primary care^a^	10.01	54.85	12.48	22.83	17.01	13.42
District hospital	7.51	10.39	18.44	9.27	11.16	8.43
Regional hospital	25.94	44.21	25.21	29.46	115.64	28.33
Overall	15.85	37.14	20.07	15.06	53.13	18.01
**Average cost of medicines and supplies used in PAC treatment**						
Primary care	9.25	0.89	22.18	44.38	21.92	11.21
District hospital	15.82	9.89	122.76	54.61	0	23.77
Regional hospital	20.40	29.49	22.56	10.06	131.67	22.26
Overall	16.22	18.79	49.67	47.42	56.97	20.20
**Average cost of hospitalization for PAC treatment**						
Primary care	0.80	5.16	3.51	3.17	10.50	1.41
District hospital	1.66	7.35	10.06	1.36	7.68	2.50
Regional hospital	8.08	6.88	10.08	6.70	10.91	8.15
Overall	4.16	6.65	8.37	2.39	8.02	4.63
**Total (by complication)**	36.23	62.58	78.11	64.87	118.02	42.84

Abbreviation: PAC, postabortion care.

a Health center and dispensary combined.

#### Medicines and Supplies

The average cost for medicines and supplies used in treating PAC cases was $20.20. However, the cost varied by complication type, from $16.22 and $18.79 for cases of incomplete abortion and shock, respectively, to $47.42, $49.67, and $56.97 for lacerations, sepsis, and perforations, respectively. These expenses varied considerably by facility type, with costs averaging $11.21 at lower-level health centers and dispensaries and increasing to $22.26 and $23.77 at regional and district hospitals, respectively. As would be expected, inputs of medicines and supplies increase with the care level of facilities, partly because more severe complications tend to be concentrated in tertiary facilities because lower-level sites frequently lack the required personnel, supplies, and infrastructure to treat critically ill patients and are thus more likely to refer them to larger facilities. This relationship may also be influenced by higher-level facilities having better access to a wider range of drugs, technology, and equipment and therefore being positioned to achieve a better standard of care than lower-level facilities.

The average cost for medicines and supplies to treat PAC was $20.20 but varied by complication type and facility type.

#### Hospitalization

Not surprisingly, the average cost per PAC client for hospitalization was highest at regional hospitals, $8.15, compared with $2.50 at district hospitals and $1.42 at lower-level health centers and dispensaries. Notably, hospitalization estimates include treatment costs associated with hospital stays over 24 hours. Basing our calculations on complication type, we found that the average cost of hospitalization ranged from $2.39 for cases of lacerated vagina or cervix to $8.02 for uterine perforation. Overall, for complications and levels of care, the average cost of hospitalization for PAC treatment among all clients who were hospitalized (n=4,092) was $7.18. Among all PAC clients in general (N=6,336), it was $4.63 (Table 4).

Overall, we found an average direct cost per case, excluding capital costs, of $42.84: 47% of this cost was attributed to medicines and supplies, 42% on personnel, and 11% on hospitalization. Management of complications was the least expensive at the primary care level, $26.04 per case, compared with $34.70 and $58.74 per case at district and regional hospitals, respectively. On average, treating incomplete abortion cost $36.23, nearly half the average cost of treating the 4 other more severe complications.

### Costs of Provision of PAC Treatment for Incomplete Abortion, Contraceptive Counseling, and Voluntary Access to Contraceptive Methods

Based on the information presented in Table 2, we estimated the comparative cost of using particular methods for treating incomplete abortion and providing family planning counseling and voluntary access to a range of contraceptive methods as a key component of PAC. Overall, the average cost per PAC client for treating incomplete abortion with either MVA, misoprostol, or sharp curettage and providing these clients with family planning counseling and voluntary access to short-acting, long-acting, or permanent methods was $35.32. Misoprostol was the least expensive treatment alternative on average ($18.74), followed by MVA ($22.63), and sharp curettage had the highest average cost ($32.02). Overall, the average cost of providing contraceptive counseling and voluntary method provision as a part of PAC was $11.56 per client, with costs varying by method: $6.23 for short-acting methods, $9.80 for LARCs (i.e., intrauterine device or implant), and $74.22 for permanent methods.

The average cost of integrating contraceptive counseling and voluntary method provision with treatment for postabortion complications was $11.56 per client.

### Indirect Costs for PAC Services

Indirect costs were estimated based on reports of approximated wages paid to all nonmedical staff and expenditures on outsourcing, maintenance, electricity and utility charges, and other assorted goods and services. Although we included these in our analysis, we acknowledge that indirect expenditure likely included additional costs that we could not account for in this estimation. Such costs would include general program management and supervision, health education, monitoring and evaluation, information systems, and management of the supply chain. For example, in the United Nations Population Fund (UNFPA) Reproductive Health Costing Tool, indirect costs are roughly twice the magnitude of direct costs in sub-Saharan Africa.^24^ In other studies, one-quarter to one-third of the total PAC costs were on indirect expenditures. Thus, we believe that our approximation should be considered as an underestimate. Nevertheless, we included them in order to determine an overall cost that is as accurate as possible. An allocative method was used to classify PAC overhead cost from the overall hospital overhead costs. The average overhead cost was $16.62. However, it varied widely by level of health care, ranging from $33.34 at regional hospitals to $5.92 and $2.09 at district hospitals and lower-level health centers and dispensaries, respectively (Box 2).

BOX 2. **Key Postabortion Care Cost Considerations**

- 18% of the unit cost of postabortion care is expended on capital costs (e.g., amortized annual costs of infrastructure and equipment), 59% on recurrent costs (personnel, medicines and supplies, hospitalization), and 23% on indirect costs.
- Treating abortion complications before they become severe (i.e., routine complications from incomplete abortion) costs on average roughly half as much as treating severe complications.
- Of the cases of routine complications from incomplete abortion, those treated with misoprostol cost on average $18.74; those treated with manual vacuum aspiration, $22.63; and those treated with sharp curettage, $32.02.
- The average cost of voluntary postabortion contraception was $11.56, roughly one-third the recurrent cost of postabortion care.

### Total Unit Cost of PAC

We calculated the total health system cost per case by adding the total costs of expenditure on capital costs, medicines and supplies, personnel, hospitalization and indirect costs and dividing the sum by the total number of PAC clients estimated to have received PAC at all study sites (N=6,336). We thus calculated a total cost of $72.91 per case. Based on our calculation, 59% of the cost was from direct costs associated with medicines and supplies, personnel, and hospitalization (i.e., medical costs), and 41% was from capital and indirect costs (i.e., nonmedical costs). Table 5 presents the health system cost per case by health care level and type of complication, and it shows the proportion of the total amount that is attributable to the different cost categories.

**TABLE 5.** Unit and Total Health System Cost of Postabortion Care at 31 Sites in Tanzania

Cost Category by Complication Type	Costs by Health Care Level (US$)				
	Regional Hospital (n=2,678)	District Hospital (n=2,178)	Lower-Level Sites^a^ (n=1,480)	All Facilities (N=6,336)	% by Category
**Personnel, total**					
Incomplete abortion	56,218.53	13,507.28	12,133.12	81,858.93	
Sepsis	5,621.06	2,378.18	1,534.56	9,533.80	
Shock	10,963.72	1,194.84	4,704.29	16,862.86	
Lacerations	530.22	1,075.32	1,210.18	2,815.72	
Uterine perforation	2,544.13	211.96	272.25	3,028.35	
Total	75,877.66	18,367.59	12,133.12	114,099.66	25%
**Medicines and supplies, total**					
Incomplete abortion	44,201.42	28,458.45	11,071.97	83 ,731.84	
Sepsis	5,030.30	15,836.12	2,728.23	23,594.65	
Shock	7,312.56	1,136.79	80.83	8,530.19	
Lacerations	181.12	6,334.45	2,351.98	8,867.55	
Uterine perforation	2,896.66	0	350.77	3,247.42	
Total	59,622.05	51,765.81	16,583.78	127,971.65	28%
**Hospitalization, total**					
Incomplete abortion	17,516.65	2,990.67	958.03	21,465.35	
Sepsis	2,248.37	1,297.29	431.74	3,977.39	
Shock	1,706.13	844.94	469.88	3,020.95	
Lacerations	120.59	157.61	168.01	446.21	
Uterine perforation	239.97	146.00	70.9	456.86	
Total	21,831.70	5,436.51	2,098.56	29,366.76	6%
**Capital costs, total**	84,304.07	604.18	301.21	85,209.47	18%
**Indirect costs, total**	89,287.79	12,901.78	3,099.08	105,288.66	23%
**Medical costs, total**	157,331.41	75,569.91	38,536.75	271,438.07	59%
**Nonmedical costs, total**	173,591.87	13,505.96	3,400.30	190,498.13	41%
**Overall total**	331,143.53	89,145.83	41,937.04	461,936.20	100%
**Total per case (N=6,336)**	**123.65**	**40.93**	**28.34**	**72.91**	

a Health centers and dispensaries combined.

### Out-of-Pocket Expenditures for PAC

Some data on OOP costs were collected, although the main purpose of this study was to evaluate how much is spent on PAC to guide systems-level planning, not who pays. Nevertheless, it is relevant to understand the proportion of these expenditures that are effectively recouped by the health system and to realize the financial burden that women and households face in seeking this service. Notably, the OOP costs presented here provide only a partial picture of this burden. Many other costs may be involved during care seeking and PAC.^25^ Table 6 reports on the average OOP costs incurred by clients when accessing PAC. Given its relevance to the overall purpose of this analysis, we present the OOP expenditure in general terms and by type of expense and rural and urban location of the facility where PAC clients received care. We did not compile information on OOP costs by complication type. The overall average OOP cost incurred by PAC clients at the surveyed facilities was $22.96. On average, PAC clients bore the most expense, roughly 21% of the total OOP cost, for medicines ($4.89). The specific costing category that represents the lowest proportion of OOP cost, 16%, was hospitalization per patient ($3.58). Differences in the level of OOP costs in urban and rural facilities apparently reflect the fact that regional hospitals, which are more likely to hospitalize PAC clients, require laboratory tests, and prescribe costly medications, are situated in urban areas.

Clients incurred on average $23 in overall out-of-pocket costs for PAC.

**TABLE 6.** Total Average Out-of-Pocket Costs for Postabortion Care by Place of Residence (US$2016)

Out-of-Pocket Costs	Urban	Rural	All
Travel cost	3.59	6.64	4.69 (20.43%)
Hospitalization	5.59	0	3.58 (15.58%)
Food	3.93	3.09	3.63 (15.81%)
Medicines	5.82	3.03	4.89 (21.30%)
Laboratory tests	4.89	1.16	3.65 (15.90%)
Other^a^	3.12	1.90	2.52 (10.98%)
Total out-of-pocket costs	26.94	15.82	22.96 (100.00%)

a Includes hospital registration fees, paying for miscellaneous drugs and supplies (e.g. gloves, Panadol), bed covers, and sheets for ultrasound.

### National Costs of PAC

Using our estimates for providing PAC from 2016, we calculated the total annual cost for treating abortion complications, including provision of voluntary postabortion contraceptive services. For this, we drew upon the conclusions of Keogh and colleagues,^17^ who reported that annually 405,000 women in Tanzania have abortions, with approximately 6 times as many women having an abortion but not receiving PAC. We also applied our estimates to other findings from the Guttmacher Institute, which estimated that annually about 40% of all women who experience complications from induced abortion receive PAC.^21^ Based on this, we estimated that 57,203 women in Tanzania received PAC in 2016, while 143,009 actually needed it. We found that the total cost incurred by the national health system on PAC provision in 2016 was $4,170,476 and that the total cost of providing PAC to all women estimated to have needed it would have been $10,426,299.

### Sensitivity Analysis

Since the data for our analysis came from different sources, mostly from key informant PAC experts, we expected a degree of uncertainty in our estimates. Consequently, we conducted a univariate sensitivity analysis of key variables to assess the impact that changes in particular parameters used to estimate unit costs would have on the output results of our unit cost analysis. This analysis tested the robustness of the models used for our estimations, focusing on the direct costs on PAC provision (medicines and supplies, personnel, and hospitalization), capital expenditures, and indirect costs. The central estimates from these data as well as the minimum and maximum estimates are presented in Table 7. Several ad hoc decisions were made regarding minimum and maximum estimates. For example, our estimates of salaries and allowances likely underestimated the actual levels of labor costs; therefore, we set the minimum estimate for labor costs at 10% less than the central estimate, and the maximum at 25% higher. However, for hospitalization, which we believe could have been overestimated as easily as underestimated, we set the minimum and maximum estimates at 20% less and greater than the central estimate, respectively. We assumed that the distribution of abortion complications could vary from the central estimates by ±15%. In addition, capital and indirect costs were assumed to have been underestimated during our study. Therefore, we set our minimum estimates at 8% of the total direct medical expenditures less than the central estimates. For our maximum estimates, we adopted the assumption reflected in the UNFPA Reproductive Health Costing Tool that indirect and capital costs amount to twice the amount of direct medical expenditure.

**TABLE 7.** Central, Minimum, and Maximum Estimates Used in Sensitivity Analysis

Parameters	Estimates	Minimum	Maximum
**% Distribution of complication**			
Incomplete abortion	81.5	72.5	100
Sepsis	7.4	3.9	11
Shock	7	4.5	11
Laceration	2.9	1	5
Uterine perforation	1	0.5	2
**Personnel**			
Total work hours for postabortion care per year	1,920	1,720	2,400
Salaries/allowances	—	10% lower	25% higher
**Hospitalization**	—	20% lower	20% higher
**Capital costs (% of direct medical costs)**	31	23	100
**Indirect costs (% of direct medical costs)**	39	31	100

Using the minimum and maximum estimates as the likely boundaries of the actual cost for PAC expended by the health system we found wider ranges when including indirect and capital costs than when accounting for the direct medical costs per intervention only. Overall, we found that, across all complications and including indirect and capital costs, the total cost of PAC to the health system per client is likely between $54.13 and $147.64 (Table 8). Expressed as the total cost to the health system, the cost is approximately $4.2 million, within a range of $3.1 and $8.4 million.

We found that the total cost of PAC to the health system per client lies between about $54 and $148.

**TABLE 8.** Sensitivity Analysis for Cost to the Health System for PAC per Case (US$)

Overall Cost	Minimum	Reported	Maximum
**Direct medical costs**			
Incomplete abortion	153,506.21	187,057.72	256,336.13
Sepsis	18,367.74	37,104.48	57,619.47
Shock	17,092.98	28,413.55	42,851.40
Lacerations	3,735.90	12,132.92	22,003.43
Perforations	3,221.23	6,729.37	13,750.38
All complications	195,924.05	271,438.04	392,560.18
**Medical cost per PAC case**	30.92	42.84	61.96
**Direct nonmedical costs**			
Capital costs	63,497.21	85,209.47	271,438.04
Indirect costs	83,573.62	105,288.66	271,438.04
Total annual overhead (capital and indirect costs)	147,070.83	190,498.13	542,876.08
**Nonmedical cost per PAC case**	23.21	30.07	85.68
**Overall cost per PAC case**	54.13	72.91	147.64

Abbreviation: PAC, postabortion care.

## DISCUSSION

In our study, conducted in 2017, we aimed to estimate the previous year’s unit and health system cost of PAC provision and offer guidance on the budget requirements for satisfying the total demand for PAC, including for those who needed PAC but could not access it. We employed data collection and analysis methods used in earlier studies for estimating PAC costs. Our methodology emphasized key informant interviews and an “ingredient approach” to costing components of the WHO Mother Baby Package, in this case the 5 major abortion complications: incomplete abortion, sepsis, shock, vaginal or cervical laceration, and perforation of the uterus or lower abdomen. We also estimated and compared the unit cost of various available methods of treatment for incomplete abortion and voluntary postabortion family planning.

We estimated that the unit cost of PAC in Tanzania stood at $72.91. Our analysis benefited from previous research that reported a 1:6 ratio of women in Tanzania who receive PAC to those who experience abortion, per year, and that women who access PAC represent 40% of those in the country who need it. We approximated that the total cost incurred by the health system for delivering PAC was $4.2 million. Given the uncertainty of some of the assumptions made in computing this estimate, we subjected the data to a sensitivity analysis, which showed that the total health system spent between $3.1 million and $8.4 million. This amount would increase to roughly $10.4 million, with a range of $7.7 to $21.1 million, if all women in the country who have abortion complications were to receive PAC.

The health system spent between $3.1 million and $8.4 million on PAC, which could increase to $10.4 million if all women needing PAC received it.

Our estimates align reasonably well with those derived through similar methodologies in neighboring countries.^11^^,^^12^ Our estimate of the direct medical cost per PAC client, $42.84, is close to estimates of $41.43 and $47.05 reported for Uganda and Rwanda, respectively. In both of these countries, the methodology derived higher overall unit costs due to differentials in the estimation of capital expenditures and indirect costs. Although our estimation for indirect costs, $16.62 per PAC client, is similar to the estimate of $18.89 in Uganda, our measure of the unit capital cost is much lower, $13.44 to $70.91. In Rwanda, whose overall unit cost estimate is closer to that derived for Tanzania, our estimate for the unit capital costs was nearly identical, $13.44 to $13.57. However, the Rwanda estimate for unit indirect costs was nearly twice our estimate for Tanzania, $32.18 to $16.62. Higher expenditures on capital costs per PAC client in Uganda arises from differences in key informants’ estimation of amounts spent on infrastructure and equipment. Key informants in Uganda also differed from those in Tanzania in terms of how they perceived the durability of PAC-related infrastructure and equipment inputs, which they conjectured had a greater number of useful life years. Estimations of indirect costs in Rwanda were nearly double those that we derived for Tanzania. As with differences between capital cost estimates between Uganda and Tanzania, key informants in Rwanda and Tanzania differed in their estimates for expenditure on annual wages for nonmedical workers at sampled facilities, the cost of outsourced contracts per facility, and annual building maintenance and operational expenses.

According to Tanzania’s Health Sector Strategic Plan IV, the government’s total budget for health for 2016 was $484,810,000.^26^ The total amount required for reproductive, maternal, newborn and child health during 2015–2016 were estimated to be $108 million.^27^ Thus, our estimates suggest that the cost of treating abortion complications in Tanzania may have been about 4% of the expenditure required for reproductive, maternal, newborn, child, and adolescent health programming. While the evidence is very limited, surveys of key informants indicate that a large proportion of women experiencing abortion complications never access care through the formal health system.^28^ In Tanzania, where this proportion is estimated to be 60%, we found that if all women who experienced abortion complications were to have received PAC, this amount would have been about 10% of the Government of Tanzania’s total expenditure on reproductive, maternal, newborn, child, and adolescent health programming. Although our estimates are based on expert opinion, not population-based data, and it is likely that women not attending health facilities may generally have less severe symptoms than those seeking treatment, it seems clear that the cost of abortion complications on the health system is appreciable.

Given what we have learned about the unmet need for PAC and the cost, health systems improvements are clearly required. We report an average OOP cost of $22.96 per woman who receives PAC, based on reports from clients who had just received the service. This OOP cost is significantly greater than those for other women’s health services, such as maternity care, which has been estimated to be $5.10 per client in Tanzania.^29^ This finding raises critical equity concerns, drawing attention to the need for pro-poor strategies to enhance the accessibility of PAC for women with limited financial means. We estimate that the cost recovered by the health system from PAC clients’ OOP expenses, excluding transportation costs, stands at $18.27, which is more than our estimate of the average indirect costs per client and approximately 25% of the overall unit cost.

Pro-poor strategies are needed to enhance the accessibility of PAC for women with limited financial means.

Reductions of the health system cost of PAC could partly be achieved by improving the availability of appropriate treatment for incomplete abortion, particularly at the primary care level. Our analysis suggests that this would occur most precipitously if vacuum aspiration and medical methods were available at this level of care. In doing so, planners could avoid the higher costs of PAC at tertiary sites, where levels of hospitalization are high and often unnecessary, and make the service more accessible to women nearer to where they reside and need care. Although misoprostol for PAC is permitted in Zanzibar, the region lacks clear policy and technical guidance on its use for PAC. In the mainland, misoprostol is not permitted for PAC. Several examples from other countries in the region demonstrate how addressing those issues enabled public health care systems to improve equity and coverage of PAC for women in need.^30^^,^^31^ Findings from a recent analysis of Service Provision Assessment data in Tanzania also suggest the need to strengthen emergency obstetric care, which includes emergency uterine evacuation as a key signal function, at lower-level health facilities, which were significantly less likely to maintain emergency obstetric care readiness.^32^ We found that contraceptive services cost $11.56 per client on average, which is less than half the cost of treating a routine abortion complication. This finding highlights the importance of investments in voluntary family planning services, not only as part of PAC but also independently to prevent unintended pregnancies in the first place.

### Limitations

This study has several limitations. First, we focused on the immediate costs incurred by the health system on treating abortion complications, and we did not address expenditures that systems accrue due to longer-term morbidities arising from complications, particularly those from unsafe abortions.^10^ Although we were unable to obtain credible estimates of longer-term abortion-related morbidity, we acknowledge that this limitation might have resulted in underestimating the total health systems cost of treating abortion complications. Furthermore, our examination of OOP used a relatively small sample size and failed to deeply explore the economic consequences of enduring abortion complications. Additional costs of this nature include opportunity costs associated with care seeking and productivity time lost due to abortion-related morbidity and mortality, all likely to severely affect the well-being of women, households, and societies.^25^^,^^33^ Not including these potential costs in our analysis likely resulted in an underestimation of the true economic consequences of resorting to PAC. Our approximations for capital and indirect costs reflect challenges faced by similar studies, which also acknowledged the difficulty of relying upon expert estimations for this measure. In our study, this factor likely resulted in an underestimation of the true capital and indirect costs related to PAC. Further, as noted in similar studies, we also emphasize the need to continue refining methods in order to capture these costs more accurately.^13^ Finally, we used data based on expert opinion, rather than population-based data, and the projections of national-level costs relied on data from a different study.

## CONCLUSIONS

Ultimately, better policy would be aimed at addressing the root cause of abortion—that is, the high incidence of unintended pregnancies, which reflects an unmet need for modern contraception among women who wish to delay, space, or limit future childbearing and who are not currently using a method. In Tanzania, approximately 1 million unintended pregnancies occur per year and 20% of women of reproductive age have an unmet need for family planning. It has been reported that 39% of all unintended pregnancies end in abortion.^34^ Our study illustrates the large burden this imposes on the Tanzanian health care system, as well as some aspects of the high costs incurred by PAC clients when they access needed care. Cost-effectiveness studies have demonstrated that increased investments in voluntary family planning services can generate net benefits by reducing the budgetary allocations required for treating complications from abortion.^35^ Nevertheless, the need for PAC to treat abortion complications can never be eliminated in a health system as it is also a key component of emergency obstetric care. Efforts to expand coverage, ensure affordability to PAC clients, and equity of access, while minimizing costs, should emphasize provision of appropriate PAC treatment and voluntary postabortion contraception, particularly at primary care levels with greater reach where the service can be introduced and sustained with minimal capital and other startup costs. The wider implications of this approach in terms of health systems strengthening should occupy a prominent place in countries’ planning and priority setting processes.

## Notes

**Peer Reviewed**

**Funding:** This manuscript was generated from a study conducted under the Post Abortion Care Project in Tanzania funded by the United States Agency for International Development under agreement AID-OAA-A-00050.

**Disclaimer:** The views expressed in this article are solely the views of the authors and do not reflect the views of the United States Agency for International Development or the United States Government.
